# MiR‐101 and doxorubicin codelivered by liposomes suppressing malignant properties of hepatocellular carcinoma

**DOI:** 10.1002/cam4.1016

**Published:** 2017-01-30

**Authors:** Fei Xu, Jia‐Zhi Liao, Guang‐Ya Xiang, Peng‐Xuan Zhao, Feng Ye, Qiu Zhao, Xing‐Xing He

**Affiliations:** ^1^ Institute of Liver DiseasesTongji HospitalTongji Medical CollegeHuazhong University of Science and TechnologyWuhanChina; ^2^ School of PharmacyTongji Medical CollegeHuazhong University of Science and TechnologyWuhanChina; ^3^ Department of PediatricsTongji HospitalTongji Medical CollegeHuazhong University of Science and TechnologyWuhanChina; ^4^ Department of GastroenterologyZhongnan Hospital of Wuhan UniversityWuhanChina

**Keywords:** Combination therapy, doxorubicin, liposome nanoparticles, liver cancer, microRNA

## Abstract

MiR‐101, an important tumor‐suppressive microRNA (miRNA) in hepatocellular carcinoma (HCC), has been affirmed significantly downregulated in HCC and participated in promoting apoptosis, decreasing proliferation and invasiveness of HCC cells, as well as increasing sensitivity of chemotherapeutic drug. However, miR‐101‐based combination therapies with doxorubicin (DOX) are not reported yet. Recently, nanomaterials‐based approaches, especially liposome formulations, have been approved for clinical use and seem to provide a great opportunity to codeliver therapeutic agents for cancer therapy. In this study, we have successfully prepared liposome (L) nanoparticles to efficiently deliver miR‐101 and DOX to HCC cells simultaneously. The effects of codelivery system miR‐101/doxorubicin liposome (miR‐101/DOX‐L) on tumor malignant phenotypes of HCC cells were evaluated through analyzing cell proliferation, colony formation, cell migration, cell invasion, cell apoptosis assay, and the expression of related genes. In subcutaneous xenografts developed by HCC cells, the inhibition of tumor growth was analyzed through gross morphology, growth curve, proliferation marker Ki‐67, apoptosis signals, and the expression of related genes. These experiments demonstrated that miR‐101/DOX‐L inhibited tumor properties of liver cancer cells in vitro and in vivo through targeting correlative genes by combinatory role of miR‐101 and DOX. In conclusion, our results indicated that liposome nanoparticle is a reliable delivery strategy to codeliver miR‐101 and DOX simultaneously, and miR‐101‐ and DOX‐based combination therapy can result in significant synergetic antitumor effects in vivo and vitro.

## Introduction

Hepatocellular carcinoma (HCC), as the major form of primary liver cancer, is a worldwide malignancy. Despite the accumulation researches of genetic and epigenetic changes in HCC, the understanding of the molecular mechanisms and therapeutic options for unresectable HCC are very limited 1. Hence, identifying new molecules for targeted therapy and developing novel treatment strategies are urgently needed.

MicroRNAs (miRNAs) are endogenous, evolutionarily conserved, small noncoding RNAs that posttranscriptionally regulate the expression of genes. Dysregulation of miRNAs appears to play fundamental roles in many cancers, and replacement of downregulated miRNAs in tumor cells may result in positive therapeutic responses. Furthermore, their small size makes them attractive for drug development. MiR‐101 has been found prevalently downregulated in multiple types of cancer, including gastric cancer, colorectal cancer, liver cancer, lung cancer, melanoma, and so on 2, 3, 4, 5, 6. We and other teams also indentified that miR‐101 is one of the most consistently low‐expressed miRNAs in HCC 7, 8. MiR‐101 can suppress cell proliferation and invasion by regulating NLK, EZH2, and STMN1 9, 10, 11; inhibit cell migration by downregulaing STMN1 and NLK 9, 10; promote cell apoptosis by modulating Mcl‐1, Rab5A, EZH2, and STMN1 8, 10, 11, 12; inhibit angiogenesis by regulating EZH2 and JunB 10, 11; and regulate cell cycle by targeting Rab5A and EZH2 10, 11, 12. Noticeably, researches recently reported that overexpression of miR‐101 could completely suppress liver tumor formation induced by c‐Myc and AKT/Ras oncogenes 13. These studies together strongly suggested an important tumor suppressor role of miR‐101 and highlighted the attractive therapeutic potential of miR‐101 in HCC.

Doxorubicin (DOX) is one of the most frequently used anticancer drugs and the front‐line chemotherapeutic options for treating patients with HCC 14. However, the clinical applications of DOX are restricted largely due to limited tissue specificity, and especially serious cardiotoxic effects resulted from the generation of free radicals and lipid peroxidation 15. Moreover, the one‐dimensional action mechanism of single drug therapy often leads to the activation of alternate pathways resulting in development of chemoresistance and tumor relapse 16, 17. Therefore, looking for targeted delivery carrier to enhance selectivity and therapeutic effectiveness, or through the combination therapy to reduce the dose of single agent to reduce systemic toxicity of anticancer drugs, becomes increasingly urgent.

Recently, nanotechnology has emerged as an important drug delivery strategy to help revolutionize the treatment of cancer. Specially, liposomes are the most used drug delivery systems and have many merits for biomedical applications. (1) Liposomes are biocompatible and can be used to deliver both hydrophilic and hydrophobic drugs for cancer therapy. (2) The periphery of liposomes can be modified to render them long circulatory lifetime and site‐specific delivery to tumor tissues 18. Such liposome‐entrapped anticancer drugs are released slowly and are less prone to degradation. This may result in lower peak plasma concentrations of free drug and reduction of acute side effects. Liposomal drug encapsulation has been found to reduce chemotherapy‐induced toxicity in many instances, and several liposome formulations have been approved for clinical use 19. However, only limited studies have been published on the liposome‐mediated codelivery of miRNA with anticancer drugs 20, 21.

**Figure 1:**
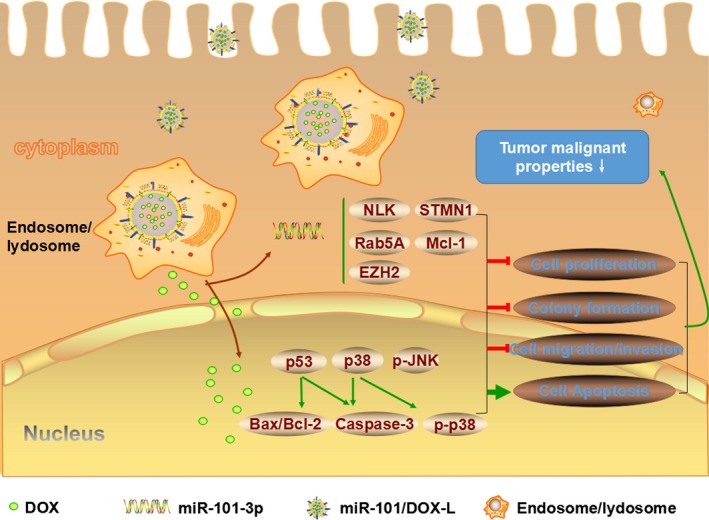
Schematic illustration of liposomes delivered miR‐101 and doxorubicin (DOX) for combination therapy in hepatocellular carcinoma (HCC). After being taken up by hepatoma cells, miR‐101/doxorubicin liposome nanoparticles escape from endosome/lysosome. Then, mature miR‐101 is released to the cytoplasm and DOX enters into nucleus. Released miR‐101 and DOX act on respective target genes and suppress tumor malignant properties of HCC cells, resulting in synergetic antitumor effects in HCC.

In the present study, we designed and prepared a liposome nanoparticle system to codeliver miR‐101 and DOX into HCC cells and tissues, and evaluated the synergetic antitumor effects of miR‐101/DOX‐L in vivo and in vitro. The research proposal of this study was presented in Figure 1.

## Materials and Methods

See Data S1 for detailed experimental methods.

### Preparation of DOX‐L and miR‐101/DOX‐L and in vitro study

The schematic diagram of design and method of this study was presented in Figure S1. Doxorubicin liposomes (DOX‐L) were synthesized by methods described in literatures previously 22. MiR‐101‐3p mimics and the negative control miR‐NC were synthesised by Guangzhou Ribobio Company (Guangzhou, China). To prepare miR‐101/DOX‐L, DOX‐L and miR‐101‐3p were mixed at w/w (weight DOX‐L/weight microRNA) ratio of more than 200:1 in RNase‐free H_2_O by adding a stock solution of DOX‐L into a miR‐101‐3p solution. The samples were vortexed for 2–3 min and then incubated at room temperature for 30 min to ensure formation of miR‐101/DOX‐L nanoparticles (Fig. S2). L, DOX‐L, and miR‐101/DOX‐L were characterized by UV‐Vis spectrophotometry and dynamic light scattering (DLS). Cellular uptake assay, TaqMan qRT‐PCR, SYBR Green qRT‐PCR, wound‐healing assay, cell proliferation, migration/invasion, apoptosis, clonogenicity assay, and western blot analysis were performed as described previously 7, and detailed in Data S1. The primer sequences used in SYBR Green qRT‐PCR are listed in Table S2.

### Animal experiments

BALB/c athymic nude mice (male, 4 weeks old) were purchased from Beijing HFK Bioscience Co., Ltd. (Beijing, China) and bred at pathogen‐free conditions. All animal experiments were carried out in accordance with the Guide for the Care and Use of Laboratory Animals of Tongji Medical College. SMMC‐7721 cells were inoculated subcutaneously into the left flank of BALB/c nude mice (4 × 10^6^ cells/mouse). Twelve days later, tumors of comparable size were established. Mice with tumor formation were randomly divided into five groups of five mice each and then the mice received intratumoral administration of normal saline, free DOX, DOX‐L, miR‐101‐L, and miR‐101/DOX‐L (2 nmol miRNA, 1 mg/kg DOX per mouse each time) respectively for four times (day 12, 16, 20, and 24). Tumor dimension was monitored every other day and their volumes were calculated by length and width using the formula: Volume = Length × Width × Width/2. Tumor tissues were collected and processed for immunohistochemical analysis. The immunohistochemistry was performed as we have described previously 23.

### Statistical analysis

Representative data from series of at least three independent experiments carried out in triplicate are presented as mean ± standard deviation (SD) unless otherwise indicated. Statistical difference between each group was assessed by unpaired two‐tailed Student's *t*‐test using Graphpad^™^ 5.0 software (GraphPad Software, Inc. La Jolla, CA, USA). Two‐tailed *P* values of less than 0.05 were considered statistically significant.

## Results

### Preparation and characterization of miR‐101/DOX‐L nanoparticles

DOX‐L and miR‐101/DOX‐L were synthesized as described in the Materials and Methods section and were characterized by DLS. By DLS detection, the average particle sizes of liposome (L), DOX‐L, and miR‐101/DOX‐L were 119.4, 121.8, and 159.8 nm, respectively. This indicated that miRNA binding to DOX‐L increased the diameter of DOX‐L by about 40 nm. The zeta potentials of blank L, DOX‐L, and miR‐101/DOX‐L were 40.6, 43.6, and 17.6 mV, respectively. The reduced zeta potential of miR‐101/DOX‐L was due to that the positive zeta potential from DOTAP was neutralized partly by the incorporation of the miRNA with a negative potential. The loading efficiencies of DOX in DOX‐L and miR‐101/DOX‐L were 87.6% and 87.8%, respectively (Table S1).

### miR‐101/DOX‐L delivers efficiently and simultaneously in HCC cells in vitro

**Figure 2:**
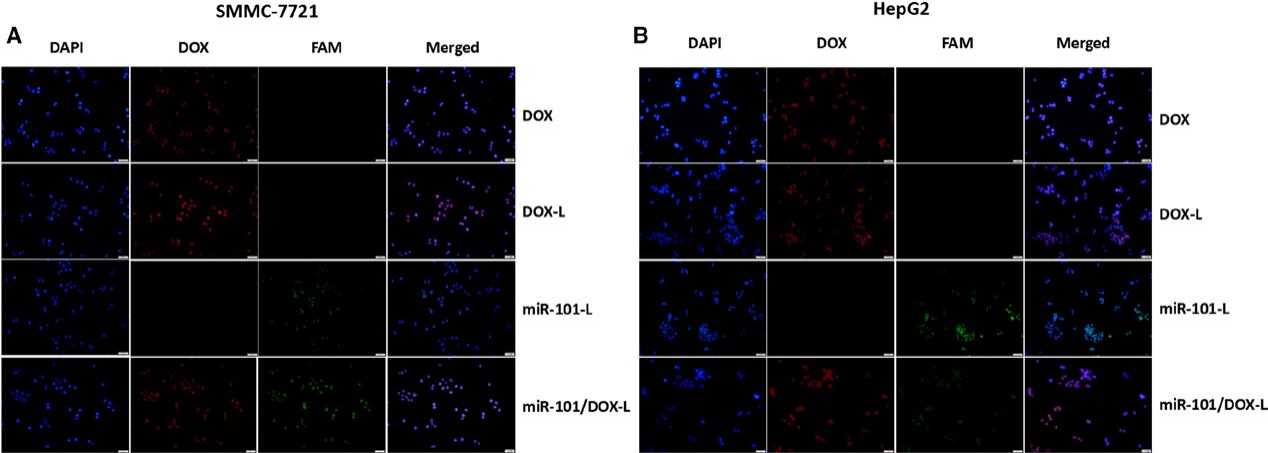
Intracellular trafficking and cellular uptake of liposome (L) nanoparticles in (A) SMMC‐7721 and (B) HepG2 cells. Cells grown in a monolayer were incubated with free doxorubicin (DOX), DOX‐L, miR‐101‐L, and miR‐101/DOX‐L for 1.5 h at 37°C. The pictures were taken under an inverted light microscope with a magnification of ×200.

SMMC‐7721 and HepG2 cells grown in a monolayer were incubated with free DOX, DOX‐L, miR‐101‐L, and miR‐101/DOX‐L for 1.5 h at 37°C. MiR‐101‐3p was labeled with FAM (Green), and DOX emits red fluorescence by itself. The nucleus was counterstained with DAPI (Blue). After 1.5 h incubation, SMMC‐7721 and HepG2 cells treated with DOX or DOX‐L showed apparent red fluorescence in almost all cells, while SMMC‐7721 and HepG2 cells treated with miR‐101‐L showed apparent green fluorescence in almost all cells, indicating an efficient and rapid uptake of DOX, DOX‐L, and miR‐101‐L by hepatoma cells, respectively (Figs. 2 and S3). Importantly, SMMC‐7721 and HepG2 cells treated with miR‐101/DOX‐L showed apparent green and red fluorescence at the same time, suggesting a quick and robust uptake of miR‐101/DOX‐L, and miR‐101 and DOX can achieve codelivery synchronously.

**Figure 3:**
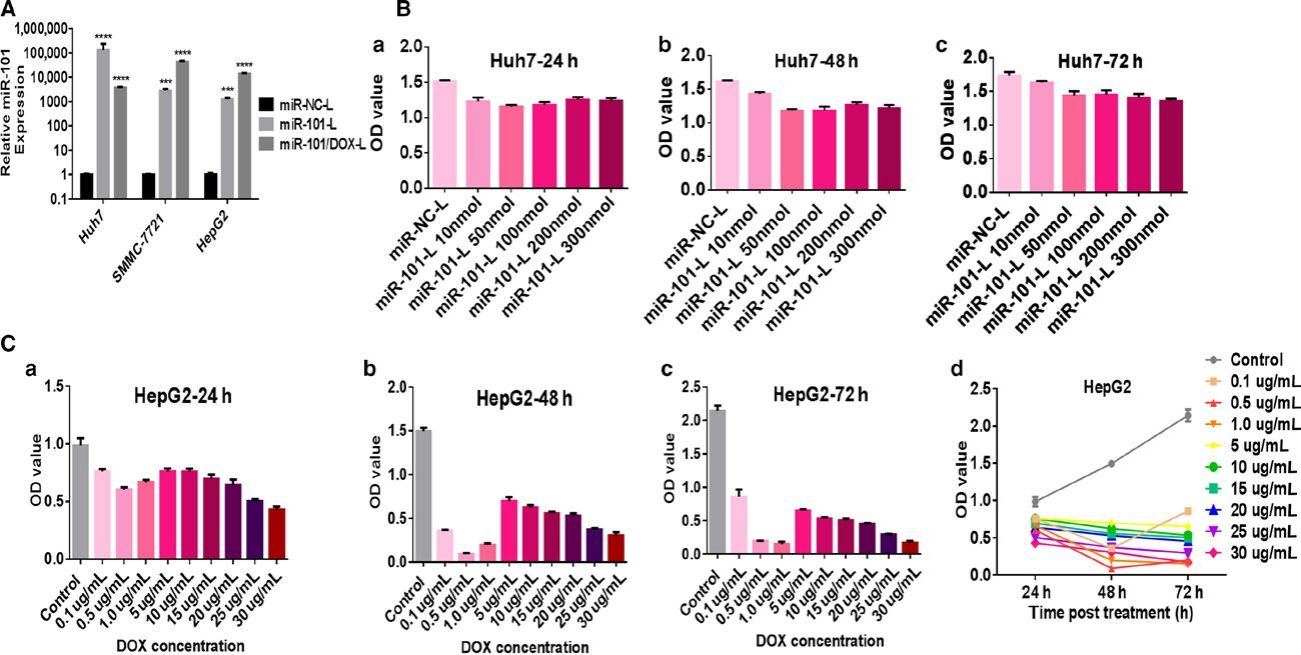
MiR‐101/DOX‐L upregulates miR‐101 effectively, and miR‐101‐L and doxorubicin (DOX) inhibit viabilities of hepatocellular carcinoma (HCC) cells in a dose‐dependent manner. (A) Quantitative analysis of the expression of miR‐101 by TaqMan qRT‐PCR in Huh7, SMMC‐7721, and HepG2 cells. The expression of miR‐101 in different HCC cells were normalized to RUN6B (mean ± SD, *n* = 3; ***P < 0.001, ****P < 0.0001). (B) Viabilities of Huh7 cells treated with different dose levels of miR‐101‐L for (a) 24, (b) 48, and (c) 72 h, respectively. (C) DOX inhibits viabilities of HCC cells effectively. Viabilities of HepG2 cells treated with different dose levels of free DOX for (a) 24, (b) 48, and (c) 72 h, respectively. (d) Trends of viabilities of HepG2 cells treated with different dose levels of free DOX over time after treatment.

Furthermore, we used TaqMan qRT‐PCR to detect the release of mature miR‐101 in hepatoma cells, and found that the expression level of miR‐101 was increased by about 4000 to nearly 50,000 times in Huh7, SMMC‐7721, and HepG2 cells treated with miR‐101/DOX‐L (Fig. 3A).

All these results suggest an efficient uptake of miR‐101/DOX‐L in HCC cells and the intact release of mature miR‐101.

### miR‐101/DOX‐L inhibits tumor properties of liver cancer cells in vitro

To optimize the synergetic antitumor effects of miR‐101/DOX‐L, cell proliferation assay was performed for miR‐101 and DOX separately with various concentrations using a CCK‐8‐based growth detection kit. As shown in Figure 3B, upregulation of miR‐101 inhibited the viabilities of Huh‐7 cells in a slightly dose–effect relationship and 100 nmol/L miR‐101‐L was chosen as our favorable concentration. The peak of the inhibitive effect of DOX on the viabilities was among 0.5–1 *μ*g/mL when the concentration of DOX was low (<5 *μ*g/mL); while cells were treated at the dose of more than 5 *μ*g/mL, DOX significantly inhibited the growth of HepG2 cells in a dose‐dependent manner (Fig. 3C). Hence, 1 *μ*g/mL DOX was chosen as our favorable concentration. These results determined the optimal concentrations of miR‐101 and DOX for our follow‐up study and suggested that rational concentrations of miR‐101/DOX‐L to HCC cells.

**Figure 4:**
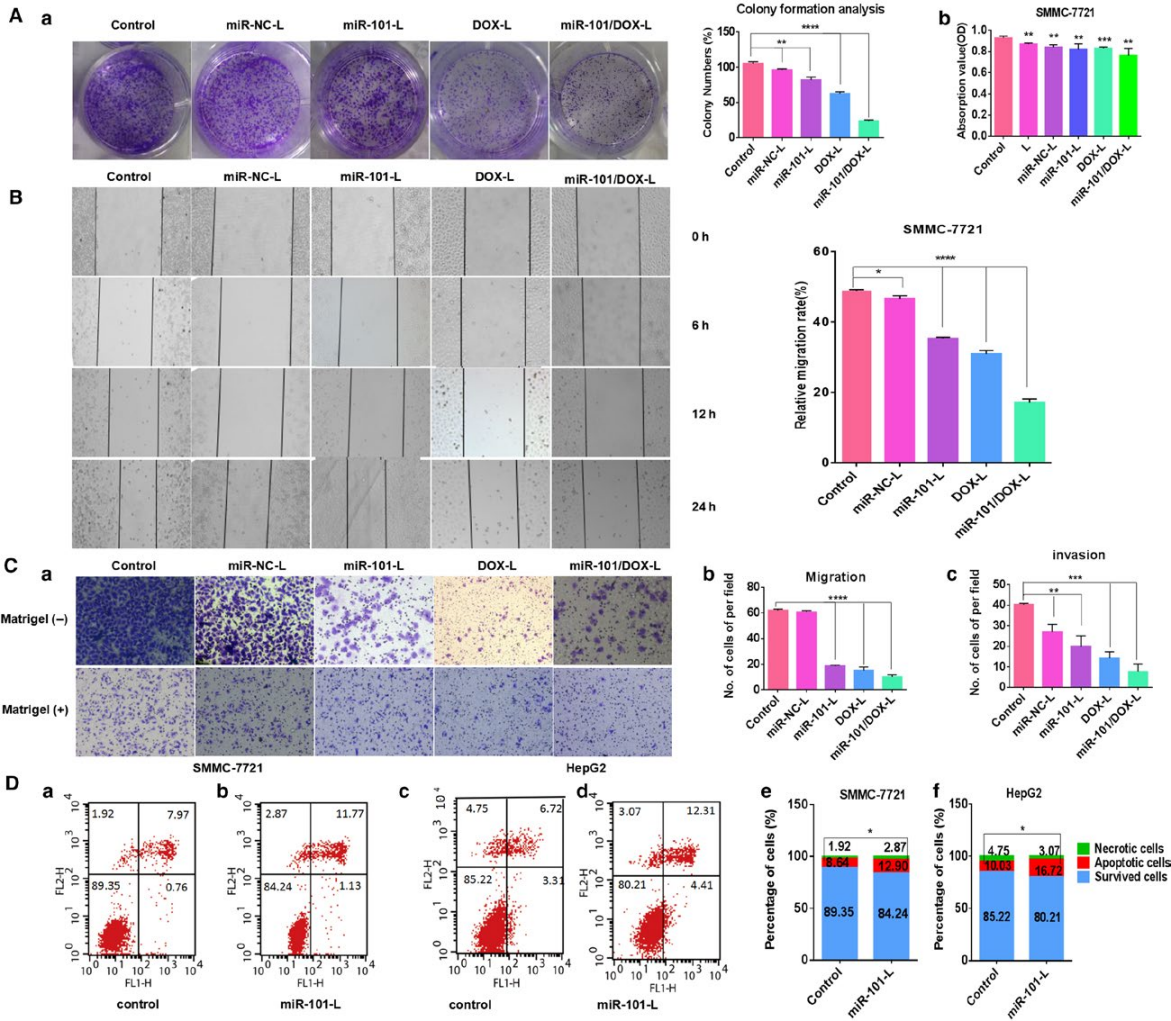
MiR‐101/DOX‐L inhibits tumor properties of liver cancer cells in vitro. Cells were treated with liposomes with the final miRNA concentration of 100 nmol/L and doxorubicin (DOX) concentration of 1 *μ*g/mL. (A) a: Plate clone formation assay was performed in SMMC‐7721 cells. Representative images and the percentages of colony numbers were shown. (A) b: Cell proliferation rate was measured by CCK8 assay at 24 h after treatment with L, miR‐NC‐L, miR‐101‐L, DOX‐L, and miR‐101/DOX‐L. (B) Cell migration of SMMC‐7721 cells was evaluated using a wound‐healing assay. Left: Cell motility was examined by light microscopy (×40) at the indicated time points (0, 6, 12, and 24 h, respectively). Right: Relative migration rate were presented in bar graph. (C) After treatment for 24 h, the cells were subjected to cell migration (upper) and invasion (lower) assays with modified Boyden chamber method. a: Representative images of the membranes of migration or invasion assay with invert microscope (×200). b, c: The cell numbers of migrated and invaded cells counted in three randomly selected microscope fields and presented in bar graph. (D) Cell apoptosis was analyzed by flow cytometry using Annexin‐V/propidium iodide combined labeling. (D) a–d: Apoptotic evaluation was determined by the percentage (%) of apoptotic cell number in total cell number. Quadrants from lower left to upper left counter clockwise represent healthy, early apoptotic, late apoptotic, and necrotic cells, respectively. (D) e, f: Cell apoptosis of SMMC‐7721 and HepG2 cell was evaluated by the percentage of apoptotic cell number in total cell number by column graph, respectively. (mean ± SD, *n* = 3; *P < 0.05, **P < 0.01, ***P < 0.001, ****P < 0.0001).

To test whether miR‐101/DOX‐L can be used for synergetic therapy in HCC, we detected phenotypic research such as clone formation ability, cell proliferation, migration/invasion, and apoptosis. Plate clone formation assay was performed to measure the cell proliferous joint effects of miR‐101/DOX‐L in SMMC‐7721 cells. Cells were treated with liposomes with the final miRNA concentration of 100 nM and DOX concentration of 1 *μ*g/mL. As shown in Figure 4Aa, the number of tumorigenic colonies was considerably impaired when cells transfected with miR‐101/DOX‐L were compared to miR‐101‐L or DOX‐L treated alone. The similar trend was confirmed by cell proliferation assay measured by CCK8 kit as presented in Figure 4Ab. Similarly, as shown in Figure 4B and C, wound‐healing and cell‐migration assays demonstrated that miR‐101/DOX‐L had an obvious inhibiting effect on SMMC‐7721 cell migration activity and the joint effect surpassed sole restoration of miR‐101. Moreover, results of Matrigel invasion assays showed that a significantly lower number of cells crossed the collagen‐coated membranes after treated with miR‐101/DOX‐L (Fig. 4C). The results appeared roughly the same as migration assay.

DOX was known to induce apoptosis. MiR‐101 can also promote apoptosis in cancer cells. To further define the role of miR‐101 on growth inhibition, we treated SMMC‐7721 and HepG2 cells with miR‐101‐L. The results showed that apoptosis in 12.9% SMMC‐7721 treated cells and 16.72% HepG2 treated cells compared with 8.64% and 10.03% in their corresponding control cells, respectively (Fig. 4D). As we expected, ectopic expression of miR‐101 caused a significant apoptosis promotion of hepatoma cells.

These results collectively confirmed that miR‐101 mimics and DOX codelivered by miR‐101/DOX‐L simultaneously can function to exert the expected phenotypic response in HCC cells and suppressed tumor properties synergistically.

### miR‐101/DOX‐L targets a cohort of genes related to miR‐101 and DOX

**Figure 5:**
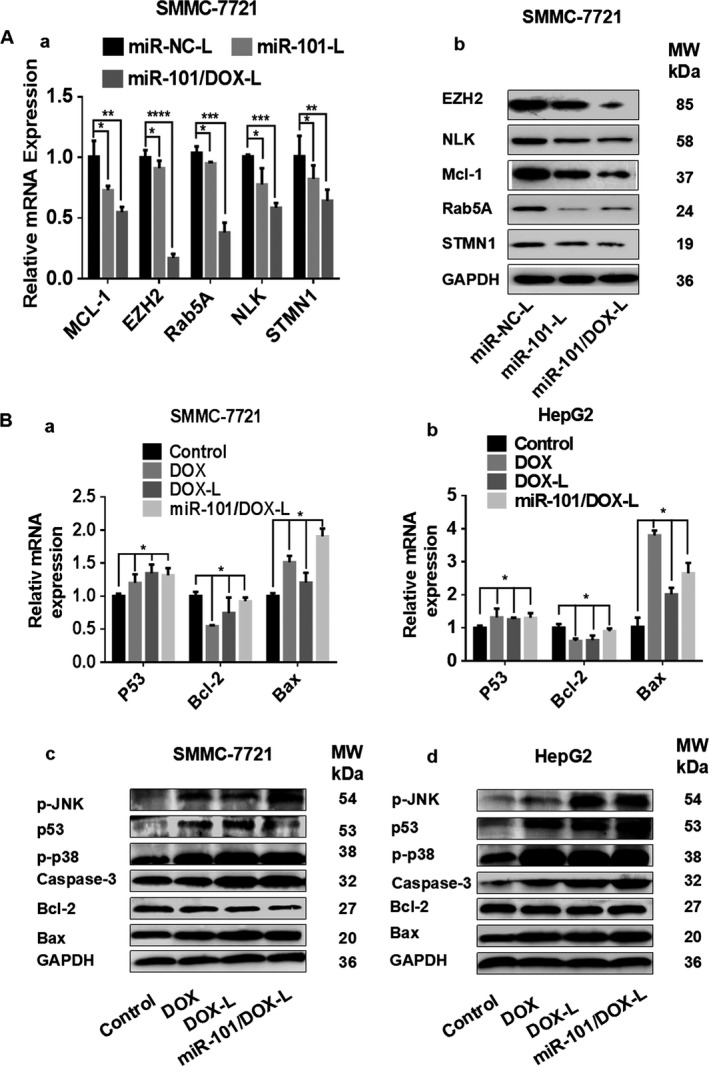
MiR‐101/DOX‐L targets a cohort of genes related to miR‐101 and doxorubicin (DOX). (A) a: The mRNA expression of Mcl‐1, EZH2, Rab5A, NLK, and STMN1 were detected by qRT‐PCR 48 h after treatment with miR‐NC‐L, miR‐101‐L, or miR‐101/DOX‐L in SMMC‐7721 cells. (A) b: The protein levels of Mcl‐1, EZH2, Rab5A, NLK, and STMN1 were detected with western blot analysis 72 h after treatment with miR‐NC‐L, miR‐101‐L, or miR‐101/DOX‐L in SMMC‐7721 cells. (B) a, b: The mRNA expression of p53, Bax, and Bcl‐2 were detected by qRT‐PCR 48 h after treatment with DOX, DOX‐L, and miR‐101/DOX‐L in SMMC‐7721 and HepG2 cells. (B) c, d: The protein levels of p53, Bax, Bcl‐2, p‐JUNK, p‐p38, and Caspase‐3 were detected with western blot 72 h after treatment with DOX, DOX‐L, and miR‐101/DOX‐L in SMMC‐7721 and HepG2 cells. GAPDH was used as a loading control.(mean ± SD, *n* = 3; *P < 0.05, **P < 0.01, ***P < 0.001, ****P < 0.0001).

NLK, EZH2, Mcl‐1, STMN1, and Rab5A have been reported as important targets of miR‐101 in HCC 9, 10, 11, 12. As shown in Figure 5A, miR‐101/DOX‐L treated group as well as miR‐101‐L group led to significant downregulation of NLK, EZH2, Mcl‐1, STMN1, and Rab5A at mRNA and protein levels compared with miR‐NC group, indicating miR‐101/DOX‐L could act on miR‐101's target genes with miR‐101's inherent mechanism.

Activated p53‐mediated promotion of apoptosis in tumor cells is an important antitumor mechanism of DOX 24. Bax, Bcl‐2, Caspase‐3 are confirmed downstream target genes of DOX in p53 signal pathway 14. Cell apoptosis is due to the makeup proportion of apoptosis protein Bax and antiapoptotic protein Bcl‐2 25. After processing SMMC‐7721 and HepG2 cells with DOX, DOX‐L, or miR‐101/DOX‐L for 48 h, the mRNA expression of is and Bax raised, whereas the mRNA expression of Bcl‐2 dropped (Fig. 5Ba and b). DOX is also found to increase the phosphorylation levels of JNK and p38 significantly and results in cell apoptosis 26. In our study, western blotting analysis showed that the protein expression of p53, Bax, p‐p38, p‐JNK, and Caspase‐3 are increased, whereas the protein expression of Bcl‐2 is decreased in miR‐101/DOX‐L‐treated SMMC‐7721 and HepG2 cells, as well as in DOX‐ or DOX‐L‐treated SMMC‐7721 and HepG2 cells (Fig. 5Bc and d).

Together, these data suggested that enhanced tumor inhibitive efficacy of miR‐101/DOX‐L resulted from the integrated suppression by releasing miR‐101 and DOX simultaneously through targeting downstream‐related genes.

### Antitumor effects of miR‐101/DOX‐L in subcutaneous xenograft model of HCC

**Figure 6:**
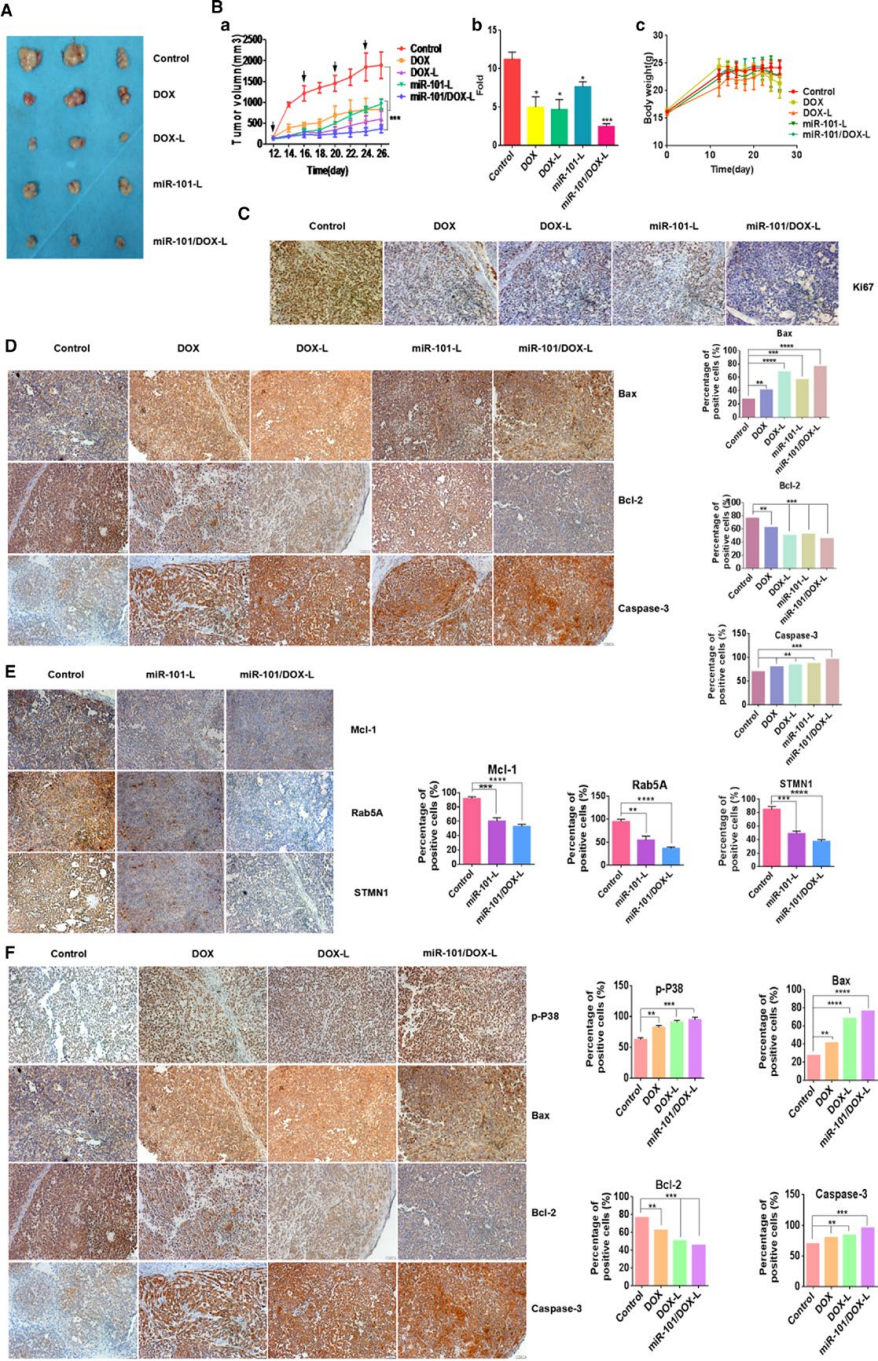
Antitumor effects of miR‐101/DOX‐L in xenograft mouse model. (A) The gross morphology of excised tumors of doxorubicin (DOX), DOX‐L, miR‐101‐L, and miR‐101/DOX‐L treated group at the end point of the experiment. (B) Growth curves of xenograft tumors. a: The average volume fold increase of tumors at the sacrifice with respect to the first measurements. b: The arrows indicated the administration of normal saline, free DOX, DOX‐L, miR‐101‐L, and miR‐101/DOX‐L per mouse every 4 days in four doses given by intratumor injection. The curves present the changes of tumor sizes from the day of injection. The differences between two groups became divergent on day 20 and this trend became more obvious and continued to the end of the experiment. The comparisons were performed versus the control group by the Student's *t*‐test. c: Body weight changes of mice treated with saline, free DOX, DOX‐L, miR‐101‐L, and miR‐101/DOX‐L. Saline injection was used as the control. (C) Immunohistochemistry staining of the proliferation marker Ki‐67 in treated tumor tissues. (D) Immunohistochemistry staining of the cell apoptosis marker Bax, Bcl‐2, and Caspase‐3 in treated tumor tissues. (E) Immunohistochemistry staining of miR‐101's target genes Mcl‐1, Rab5A, and STMN1 in treated tumor tissues. (F) Immunohistochemistry staining of DOX's effective molecules p‐p38, Bax, Bcl‐2, and Caspase‐3 in treated tumor tissues. Representative immunohistochemical staining and the percentages of positive cells were shown. Positive cells were counted in tumor tissues and presented as the mean ± SD (five random fields per section and four sections per tumor). *P < 0.05, **P < 0.01, ***P < 0.001, ****P < 0.0001.

The antitumor effects of compounds were further evaluated in a SMMC‐7721 xenograft tumor model in mice. Mice with tumor formation were randomly divided into five groups of five mice each and then the mice received intratumoral administration four times upon the subcutaneous tumor grew to ~200 mm^3^. Two weeks after the first injection, mice were sacrificed and tumors were examined. Compared with the saline group, the other groups showed a marked suppression of xenograft tumors growth, which could be reflected by gross morphology (Fig. 6A). We cannot ignore that the miR‐101/DOX‐L‐treated group displayed the most obvious inhibition effect. As shown in Figure 6Ba, the growth curves of treated tumors began to show differences on day 16 and became divergent on day 20 after the inoculation of SMMC‐7721 cells in mice, and this trend became more obvious and continued to the end of the experiment. More importantly, relative to the first measurement on day 12, the average fold increase of tumor volumes at the sacrifice of miR‐101/DOX‐L‐treated group was significantly smaller than miR‐101‐ or DOX‐L‐treated group (Fig. 6Bb). The body weights of mice after drug injections showed no significant difference between groups (Fig. 6Bc).

After sacrifice of the mice, tumor tissues from xenograft tumor were collected for histological evaluation. We detected the expression of Ki67 as markers of proliferation and Ki67 level in miR‐101/DOX‐L‐treated xenograft tumors was much lower, a largely consistent inhibitive trend of proliferation compared to a gross morphological verification (Fig. 6C). To clarify the cellular mechanism of miR‐101/DOX‐L‐mediated synergetic inhibition of tumor growth, the expression of several relative genes was measured by immunohistochemistry in tumor tissues. As shown in Figure 6D, miR‐101/DOX‐L induced the expression of Caspase‐3 and suppressed the expression of the apoptosis‐suppressing gene Bcl‐2, while simultaneously stimulated the expression of Bax which encodes a dominant inhibitor of the Bcl‐2 protein. Furthermore, Mcl‐1, Rab5A, STMN1, and Bcl‐2 protein expression were remarkably decreased while Bax, Caspase‐3, and p‐p38 expression was significantly increased at the same time in miR‐101/DOX‐L group (Fig. 6E and F).

All these data demonstrated the therapeutic effects of miR‐101/DOX‐L showed significantly higher efficacy than sole treatment in vivo, suggesting again that miR‐101/DOX‐L inhibited tumor properties of HCC through targeting correlative genes by combinatory role of miR‐101 and DOX.

## Discussion and Conclusion

MiRNA–chemotherapeutic drug combinations have been found to be effective against different molecular targets and can increase the sensitization of cancer cells to therapy several folds 27. DOX is highly effective with broad spectrum anticancer drug. However, the clinical uses of it are restricted largely due to limited tissue specificity and serious cardiotoxic effects. MiR‐101 has been found significantly downregulated and functions as an important tumor suppressor in HCC which stands for an attractive target for HCC treatment due to its capacity to inhibit tumor cell growth in vitro and in vivo 8, 10, 11, 28, 29. However, miR‐101/DOX‐L‐based combination therapy in HCC has not been reported before.

Recently, nanotechnology has emerged as an important delivery strategy for drug combination therapy in cancer. Among the nanoparticles, liposome formulations, particularly the long‐circulating pegylated class, have gained clinical recognition as an effective cardioprotective approach that maintains or even enhances the antitumor activity of DOX 15. Furthermore, several studies showed higher selective tumor localization of DOX‐L than that of free DOX 30, 31. Thus, lipid‐coated DOX nanoparticles codelivered with miR‐101 seems to be a promising therapy option with high safety index and tumor targeting.

In this article, liposomes were synthesized as expected, the size of DOX‐ and miR‐101‐encapsulated liposomes were around 150 nm. Analysis using fluorescence microscope and TaqMan qRT‐PCR showed that miR‐101/DOX‐L was taken up by HCC cells efficiently and mature miR‐101 was released without degradation. Functional analysis revealed that miR‐101/DOX‐L suppressed multiple malignant phenotypes (inhibiting proliferation, clone formation, migration/invasion, and inducing apoptosis) of HCC cells synergistically by releasing miR‐101 and DOX simultaneously through targeting downstream‐related genes, both at transcriptional and posttranscriptional levels. Codelivery of miR‐101/DOX‐L significantly enhanced its inhibitive capability compared to naked counterpart. Furthermore, our results from xenograft tumor mouse model also indicate that codelivery of DOX and miR‐101 by cationic liposome suppressed tumorigenicity of liver cancer cells effectively in vivo and the enhanced tumor inhibitive efficacy of miR‐101/DOX‐L was resulted from the integrated suppression from miR‐101‐ and DOX‐related targets. What deserved mentioned was that the body weights of treated mice showed no significant difference compared with that of control mice. Our data showed that liposome‐coated DOX nanoparticles codelivered with miR‐101 did not increase systemic toxicity, which was supported by a meta‐analysis that liposomal compared with conventional DOX significantly decreased the risk of clinical and subclinical cardiotoxicity 32.

Importantly, our data validated the rational of codelivery of DOX and miR‐101 since the selection of chemotherapeutics and gene‐related drugs were critically important for combined therapy. Both in vitro and in vivo results illustrated that these two drugs showed additive or enhanced effects, thereby providing novel targets for therapeutic intervention in liver cancer.

Clear evidences are given by the recent reports that combination delivery of miRNA and drug using nanoparticles are indeed helpful in inhibiting the tumor growth compared to miRNA or drug alone. Chen et al. developed liposome–polycation–hyaluronic acid (LPH) nanoparticle formulation modified with GC4 single‐chain variable fragment that target tumor sphere cells, a tumor‐targeting human monoclonal antibody for systemic delivery of siRNA and miRNA into experimental lung metastasis of murine B16F10 melanoma model. Results suggested that the combination of siRNAs and miR‐34a delivery by GC4‐targeted nanoparticles additively inhibited tumor growth as the tumor load decreased to about 20% compared to 30% and 50% when treated with siRNAs and miR‐34a alone 20. These studies, together with our study, collectively suggest that the synergetic or combined effect by codelivering chemotherapeutic drugs and miRNA to cancer cells is a general phenomenon and hold promise for the future personalized cancer treatments.

In conclusion, our findings demonstrated that miR‐101/DOX‐L inhibited tumor properties of liver cancer cells in vitro and in vivo through targeting correlative genes by combinatory role of miR‐101 and DOX. It is anticipated that this article provide base to further develop miR‐101‐ and DOX‐based combination therapy for HCC.

## Conflict of Interest

None declared.

## Supporting information


**Table S1.** Physicochemical properties of DOX‐L and miR‐101/DOX‐L.
**Table S2.** Primers used in SYBR Green qRT‐PCR.
**Figure S1.** Schematic diagram of project design and method of this study.
**Figure S2.** Schematic representation of cationic solid lipid nanoparticles complexed with DOX and miR‐101‐3p. (A) Empty solid lipid nanoparticles. (B) DOX‐loaded solid lipid nanoparticles. (C) Conjugation with miR‐101‐3p by DOX lipid nanoparticles.
**Figure S3.** Intracellular trafficking and cellular uptake of liposome (L) nanoparticles in SMMC‐7721 and HepG2 cells. Cells grown in a monolayer were incubated with (A) free DOX, (B) DOX‐L, (C) miR‐101‐L, and (D) miR‐101/DOX‐L for 1.5 h at 37°C. The pictures were taken under an inverted light microscope with a magnification of ×100 and ×400 respectively.
**Data S1.** Supporting materials and methods.
