# Supplementary material for: MiR‐101 and doxorubicin codelivered by liposomes suppressing malignant properties of hepatocellular carcinoma
Source: Cancer Med. 2017 Jan 30;6(3):651–61. doi: 10.1002/cam4.1016 (PMC5345655; doi:10.1002/cam4.1016)
Supplement: Supplementary file 1 — Table S1. Physicochemical properties of DOX‐L and miR‐101/DOX‐L. Table S2. Primers used in SYBR Green qRT‐PCR. Figure S1. Schematic diagram of project design and method of this study. Figure S2. Schematic representation of cationic solid lipid nanoparticles complexed with DOX and miR‐101‐3p. (A) Empty solid lipid nanoparticles. (B) DOX‐loaded solid lipid nanoparticles. (C) Conjugation with miR‐101‐3p by DOX lipid nanoparticles. Figure S3. Intracellular trafficking and cellular uptake of liposome (L) nanoparticles in SMMC‐7721 and HepG2 cells. Cells grown in a monolayer were incubated with (A) free DOX, (B) DOX‐L, (C) miR‐101‐L, and (D) miR‐101/DOX‐L for 1.5 h at 37°C. The pictures were taken under an inverted light microscope with a magnification of ×100 and ×400 respectively. Data S1. Supporting materials and methods. [file CAM4-6-651-s001.doc]

**Supporting information**

**MiR-101 and doxorubicin co-delivered by liposomes suppressing malignant properties of hepatocellular carcinoma**

Fei Xu, Jia-Zhi Liao, Guang-Ya Xiang, Peng-Xuan Zhao, Feng Ye, Qiu Zhao, Xing-Xing He

**Supporting Tables**

**Table S1. Physicochemical properties of DOX-L and miR-101/DOX-L.** Complexation of miRNA with DOX liposomes (DOX-L) and characterization of liposomes. Key parameters of L, DOX-L, and miR-101/DOX-L liposomes. Data are expressed as mean ± SD of three independent samples.

| Liposome | Particle size  (nm) | Zeta potential  (mV) | DOX Entrapment  (%) |
| --- | --- | --- | --- |
| L | 119.4±0.6 | 40.6±3.27 | --- |
| DOX-L | 121.8±0.6 | 43.6±3.04 | 87.6 |
| miR-101/DOX-L | 159.8±0.9 | 17.6±6.07 | 87.8 |

**Table S2. Primers used in SYBR Green qRT-PCR.**

**Supporting Figures**

| **Primer name** | |  | **Primer sequence** |
| --- | --- | --- | --- |
| NLK | Forward | | 5’-CTTTGAGCCTGTCACCAATCCCAA-3’ |
|  | Reverse | | 5’-CTTCCACCATCACTCCCACACCA-3’ |
| GAPDH | Forward | | 5’-ACAACTTTGGTATCGTGGAAGG-3 |
|  | Reverse | | 5’- GCCATCACGCCACAGTTTC-3’ |
| EZH2 | Forward | | 5’-TGCAGTTGCTTCAGTACCCATAAT-3’ |
|  | Reverse | | 5’-ATCCCCGTGTACTTTCCCATCATAAT-3’ |
| STMN1 | Forward | | 5’-CTCGGACTGAGCAGGACTTTC-3’ |
|  | Reverse | | 5’-GCACGCTTCTCCAGTTCTTTC-3’ |
| Mcl-1 | Forward | | 5’-TGCTTCGGAAACTGGACATCA-3’ |
|  | Reverse | | 5’- TAGCCACAAAGGCACCAAAAG-3’ |
| Rab5A | Forward | | 5’-AGACCCAACGGGCCAAATAC-3’ |
|  | Reverse | | 5’-GCCCCAATGGTACTCTCTTGAA-3’ |
| Bax | Forward | | 5’-ACCAAGGTGCCGGAACTG-3’ |
|  | Reverse | | 5’-GCACTCCCGCCACAAAGAT-3’ |
| Bcl-2 | Forward | | 5’-TTGTTTCTTGAAGGTTTCCTCGTC-3’ |
|  | Reverse | | 5’-GGGCTCTGGGTGGGTCTGT-3’ |
| p53 | Forward | | 5’-CAGCACATGACGGAGGTTGT-3’ |
|  | Reverse | | 5’-TCATCCAAATACTCCACACGC-3’ |

**
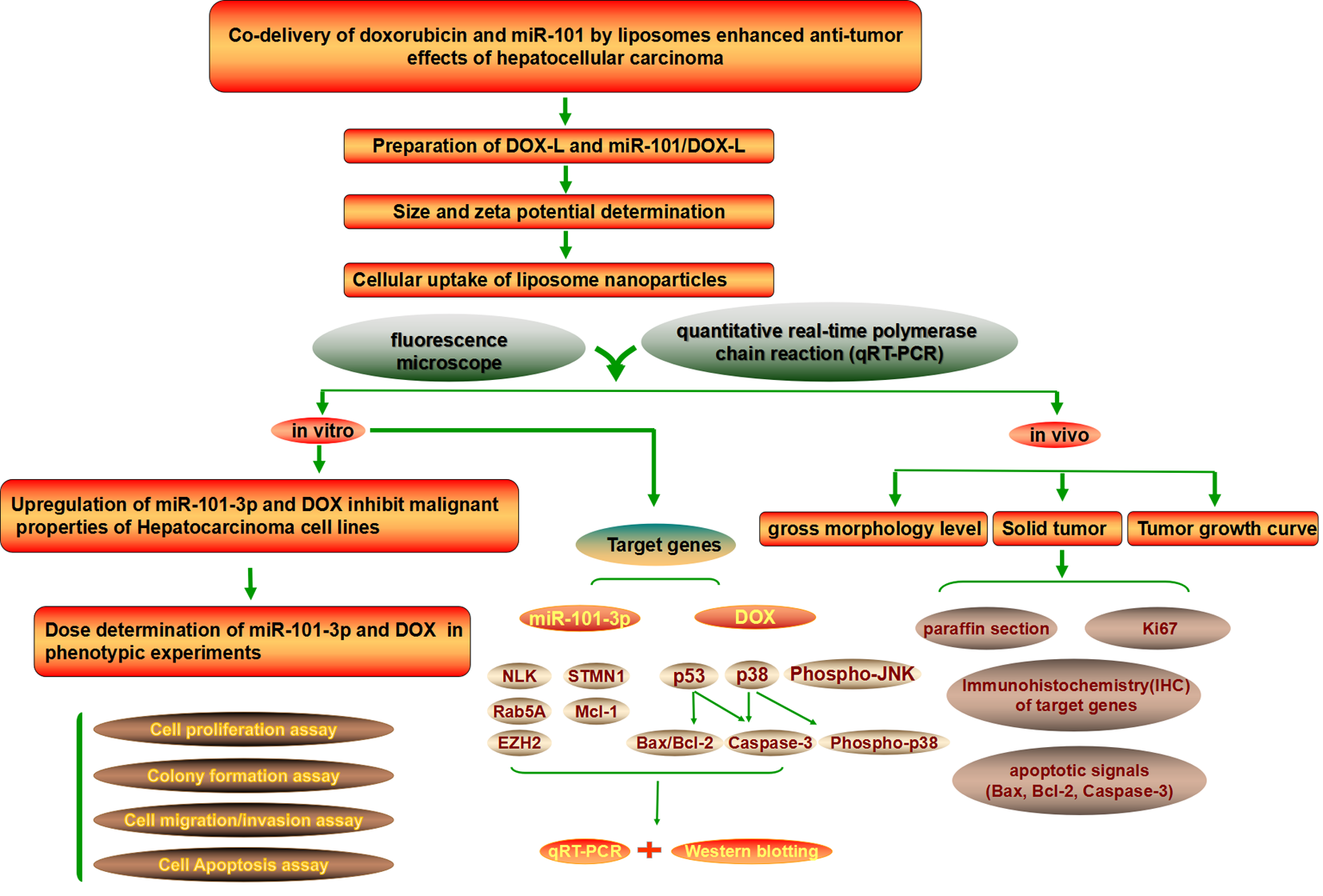
**

**Figure S1.** Schematic diagram of project design and method of this study.


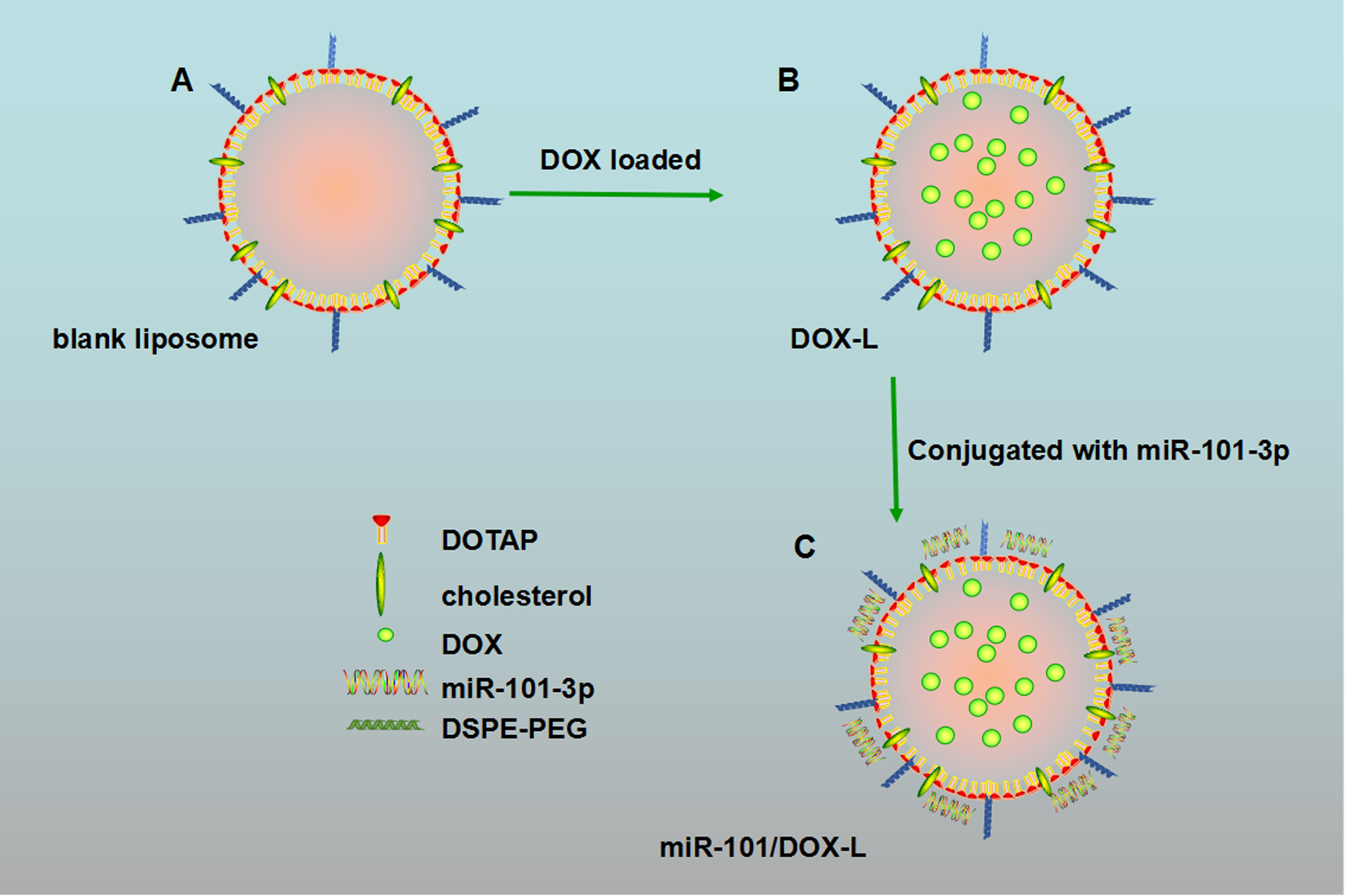


**Figure S2.** Schematic representation of cationic solid lipid nanoparticles complexed with DOX and miR-101-3p. A. Empty solid lipid nanoparticles B. DOX loaded solid lipid nanoparticles. C. Conjugation with miR-101-3p by DOX lipid nanoparticles.


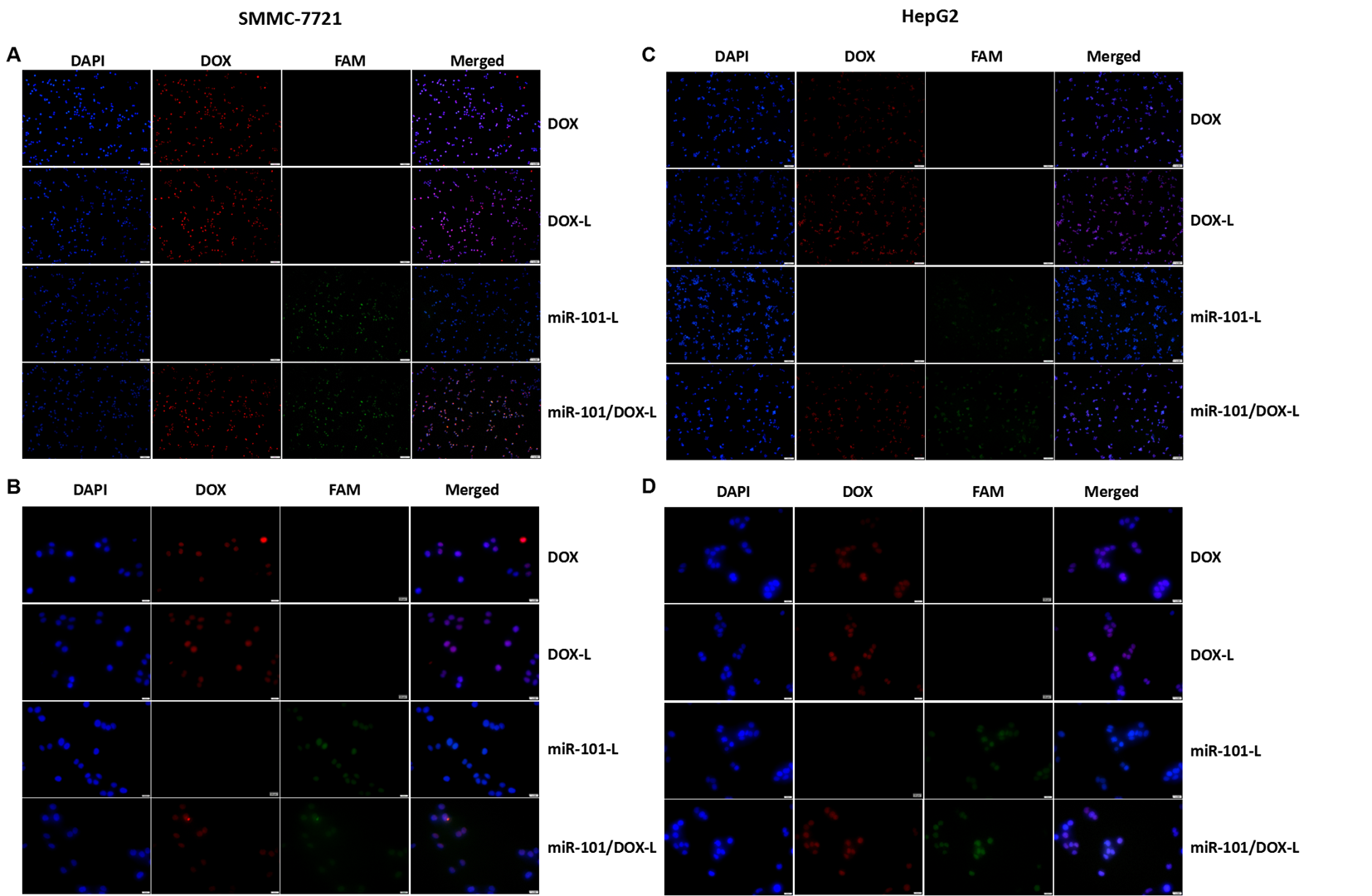


**Figure S3.** Intracellular trafficking and cellular uptake of liposome (L) nanoparticles in SMMC-7721 and HepG2 cells. Cells grown in a monolayer were incubated with free DOX, DOX-L, miR-101-L and miR-101/DOX-L for 1.5 h at 37℃. A-D. The pictures were taken under an inverted light microscope with a magnification of ×100, ×400 rerspectively.

**Supporting materials and methods**

**Chemicals for preparation of liposomes**

The schematic diagram of project design and method of this study was presented in **Supporting Figure S1**. Doxorubicin hydrochloride (DOX·HCl), dicyclohexylcarbodiimide (DCC), N-hydroxysuccinimide (NHS), cholesterol, DMSO, (NH4)2SO4, CHCl3 and Sepharose CL-4B chromatography media were purchased from Sigma-Aldrich Chemical Co (St. Louis, MO, USA). DMEM high glucose medium was purchased from the Hyclone of Thermo Scientific (IL, USA). Trypsin was purchased from Beyotime Institute of Biotechnology (Beijing, China). 1, 2-Dioleoyl-3-Trimethylammonium-Propane (Chloride Salt) (DOTAP), monomethoxy polyethylene glycol 2000-distearoyl phosphatidylethanolamine (mPEGDSPE) and PEG-bis-amine (3350 Da) were purchased from Avanti Polar Lipid (Alabaster, AL, USA). PD-10 desalting columns were purchased from GE Healthcare Biosciences (PA, USA). All reagents and solvents were of analytical or HPLC grade and were used without further purification. Negative control RNA duplexes (miR-NC) and miR-101-3p (sense strand: 5'-UCA UUU UUC GGU UAU CAU GGU ACC GAUG-3'; antisense strand: 5'-GUA CAG UAC UGU GAU AAC UGA AGA AUGG-3') were synthesized by Ribobio Co., Ltd (Guangzhou, China).

**Preparation of DOX-L and miR-101/DOX-L**

Doxorubicin liposome (DOX-L) were synthesized by methods described in literatures . The lipid compositions of liposomes were DOTAP/Chol/mPEG-DSPE at a molar ratio of 40:55:5 respectively. DOX was remotely loaded into the liposomes by a transmembrane pH gradient. Briefly, the total lipids (85 mg) was dissolved in CHCl3 and dried to a thin film by rotary evaporation followed by further drying under vacuum at 40℃. The lipid film was then hydrated with 2 mL (NH4)2SO4 (250 mM) for 30 min at 60℃. The resultant of multilamellar vesicles were extruded 5 times through 0.2 µm pore-size polycarbonate membranes and 5 times through 0.1 µm pore-size polycarbonate membranes using a Lipex Extruder (Northern Lipids Inc., Canada) driven by pressurized nitrogen at 60℃ to produce homogeneous unilamellar vesicles. The residual (NH4)2SO4 was removed with phosphate-buffered saline (PBS, pH 7.4) by size-exclusion chromatography on a PD-10 column. DOX·HCl dissolved in deionized H2O (10 mg/mL) was added to the liposomes at a DOX-to-lipid ratio of 1:20 (w/w), followed by a 30 min incubation at 65℃. Free DOX was separated from liposomes by size exclusion chromatography on a Sepharose CL-4B column. To prepare miR-101/DOX-L, DOX-L and miR-101-3p were mixed at w/w (weight NPC/weight microRNA) ratio of more than 200:1 in RNase free H_2_O by adding a stock solution of DOX-L into a miR-101-3p solution. The samples were vortexed 2-3 min and then incubated at room temperature for 30 min to ensure formation of miR-101/DOX-L nanoparticles (**Supporting Figure S2**).

**Characterization of miR-101/DOX-L**

Particle size and zeta potential of miR-101/DOX-L were measured by Zeta PALS (Zeta Potential Analyzer, Brookhaven Instruments Corporation, Austin, TX) according to the manufacturer's instructions. All measurements were carried out at room temperature. Each parameter was measured 3 times. Average values and standard deviations were calculated. The drug-loading capacity was measured using high performance liquid chromatography. Samples were digested in 70% HNO3 and diluted in water to a final acid content of 2%. A standard curve was derived from a series of dilutions of doxorubicin solutions and concentration was determined according to the standard curve.

**Cell culture**

The human HCC cell lines SMMC-7721, HepG2, and Huh7 were from the China Center for Type Culture Collection at Wuhan University (Wuhan, China) and cultured and maintained in Dulbecco’s modified Eagle medium (DMEM) supplemented with 100 ml/l fetal bovine serum（FBS, Sigma, St. Louis, MO) and 100 units/ml of penicillin–streptomycin (Invitrogen, Carlsbad, CA). All cells were cultured at 37℃ in a humidified incubator with 5% CO2.

**Cellular uptake of liposomes**

SMMC-7721, HepG2 cells grown in a monolayer were washed once with DMEM and then were incubated with free DOX, DOX-L, miR-101-L and miR-101/DOX-L at 37°C. After treatment for 1.5 h, the cells were rinsed with PBS for 3 times and fixed with 4% paraformaldehyde solution for 15 min. The solution of 4, 6-Diamidino-2-phenylindole (DAPI) was employed for the staining of nucleus. Samples were observed with a fluorescence microscope (Olympus, Japan).

**TaqMan qRT-PCR**

The expression of mature miR-101 was assayed using the TaqMan MicroRNA Assays (Applied Biosystems, Foster City, CA, USA) specific for miR-101-3p mimics and RUN6B (Applied Biosystems) according to the manufacturer’s instruction.

**Cell proliferation assay**

Cell proliferation was determined using the CCK8 (Roche Applied Science, Mannheim, Germany) according to the manufacturer’s protocol.

**Cell migration/invasion, apoptosis and clonogenicity assay**

These assays were detected as we previously described .

**Wound healing assay**

SMMC-7721 cells were incubated in 6-well plates, 24 h after treatment, clones were grown to confluency. A linear wound was made by scraping a non-opening Pasteur pipette across the conﬂuent cell layer. Cells were washed twice to remove detached cell sanddebris. Then, size of wounds were observed and measured at the indicated time points（0h, 6h, 12h, 24h rerspectively.

**RNA isolation and SYBR Green qRT–PCR**

SMMC-7721 and HepG2 cells were harvested for RNA extraction 48 h after treatment. The First Strand cDNA Synthesis Kit (Fermentas, Burlington, VT, USA) was applied to synthesize the first strand cDNA followed by real-time PCR using the SYBR Premix Ex Taq (Takara Bio Inc., Otsu, Japan). GAPDH RNA was used as a control. The 2^-△△CT^ method was used to determine relative gene expression. The primer sequences are listed in **Supporting Table S2**.

**Western blot analysis**

SMMC-7721 and HepG2 cells were harvested for protein extraction 72 h after treatment. Cell protein lysates were used for western blot analysis as described before. The following antibodies were used: EZH2 (AB110646，Abcam, Cambridge, MA, USA), NLK (AB154199，Abcam, Cambridge, MA, USA), STMN1 (AB52630，Abcam, Cambridge, MA, USA), Rab5A ( AB109534，Abcam, Cambridge, MA, USA), Mcl-1 (AB32087，Abcam, Cambridge, MA, USA), Bax (60267-1-Ig, Protein Tech, Chicago, IL, USA), Bcl-2 ((PA-001-Bcl-2, Promotor, wuhan, China), Caspase-3（AB 184787，Abcam, Cambridge, MA, USA）, and Phospho-p38 (4511S, Cell Signaling Technology, USA), Phospho-SAPK/JNK (4668S, Cell Signaling Technology, USA), p53 (PA-001-P53, Promotor, wuhan, China). GAPDH, (sc-47778, Santa Cruz, CA, USA), goat anti-mouse IgG-horseradish peroxidase (sc-2005, Santa Cruz) and goat anti-rabbit IgG-horseradish peroxidase (sc-2004, Santa Cruz).

**Animal Experiments**

BALB/c athymic nude mice (male, 4 weeks old) were purchased from Beijing HFK Bioscience Co.LTD (Beijing, China) and bred at pathogen-free conditions. SMMC-7721 cells were inoculated subcutaneously into the left flank of BALB/c nude mice (4×10^6^ cells/mouse). 12 days later, tumors of comparable size were established. Mice with tumor formation were randomly divided into five groups of five mice each and then the mice received intratumoral administration of normal saline, free DOX, DOX-L, miR-101-L, and miR-101/DOX-L (2nmol miRNA, 1 mg/kg DOX per mouse each time) perspectively for four times (day 12, 16, 20, and 24). Tumor dimension was monitored every other day and their volumes were calculated by length and width by using the formula: volume = length ×width × width/2. Tumor tissues were collected and processed for immunohistochemical analysis. The immunohistochemistry was performed as we have described before .

**Ethic statement**
The study was approved by the local research ethics committee at the Tongji Hospital of Huazhong University of Science and Technology. The methods used in this study were carried out in accordance with the approved guidelines.

**Statistical Analysis**

Representative data from series of at least three independent experiments carried out in triplicate are presented as mean ± standard deviation (SD) unless otherwise indicated. Statistical difference between each group was assessed by unpaired two-tailed Student t-test using Graphpad™ 5.0 software. Two-tailed p values of less than 0.05 were considered statistically significant.

**References**

1. YANG T, LI B, QI S, et al. Co-delivery of doxorubicin and Bmi1 siRNA by folate receptor targeted liposomes exhibits enhanced anti-tumor effects in vitro and in vivo. Theranostics 2014; 4(11): 1096-111.

2. HE X X, CHANG Y, MENG F Y, et al. MicroRNA-375 targets AEG-1 in hepatocellular carcinoma and suppresses liver cancer cell growth in vitro and in vivo. Oncogene 2012; 31(28): 3357-69.

3. YAN J J, ZHANG Y N, LIAO J Z, et al. MiR-497 suppresses angiogenesis and metastasis of hepatocellular carcinoma by inhibiting VEGFA and AEG-1. Oncotarget 2015; 6(30): 29527-42.
